# Rare case of syphilis presenting with labial chancre

**DOI:** 10.11604/pamj.2021.39.31.27959

**Published:** 2021-05-11

**Authors:** Surya Besant Natarajan, Krishna Prasanth Baalann

**Affiliations:** 1 Department of Community Medicine, Sree Balaji Medical College and Hospital, Chennai, India,; 2 Bharath Institute of Higher Education and Research, Chennai, India

**Keywords:** Sexually transmitted diseases, chancre, syphilis

## Image in medicine

Syphilis is a chronic inflammatory disease which is caused by the spirochete *Treponema pallidum* (*T. pallidum*) and is often sexually transmitted. The primary stage in syphilis classically presents with a painless ulcer which is evident in the genital area in more than 90% of patients and is commonly called as chancre. Extra genital chancres may differ from the classic ones based on the portal of entry of the organism, in terms of localization, size, depth, base and edges thus, they may cause diagnostic problems. A 27-year-old male presented with a 2-week history of a painless, ulcerative lesion on his lower lip. He gave a history of multiple sexual exposures and unprotected sex. On examination, he had a 2 x 2 cm nodular ulcer on his lower lip, palpable non-tender submental lymphadenopathy over the left side of the neck. Venereal Disease Research Laboratory (VDRL) test was reactive at 30 dilutions and *T. pallidum* hemagglutination (TPHA) test was positive. A fluorescent antibody test for *T. pallidum* showed positive for *T. pallidum* specific IgG and IgM. HIV testing was negative. Syphilis was confirmed, and findings were consistent with both primary (oral chancre and lymphadenopathy) and secondary (macular rash) disease. For treatment, the patient was given Benzathine Penicillin in 2.4 million units intramuscularly weekly once for three continuous weeks.

**Figure 1:**
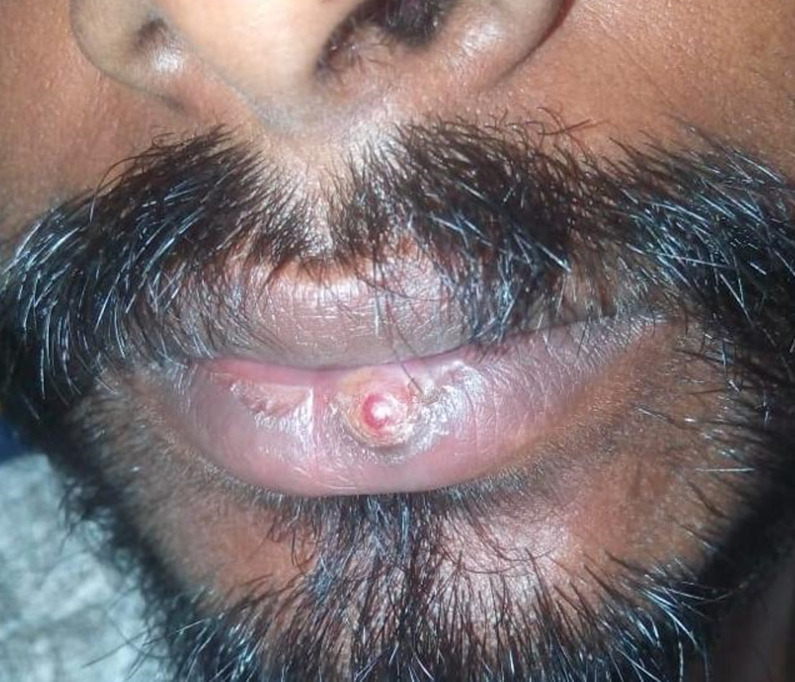
nodular ulcer on lower lip

